# Psychological predictors of music performance anxiety among vocal students: a gender-based SEM analysis

**DOI:** 10.3389/fpsyg.2025.1667730

**Published:** 2026-02-02

**Authors:** Ye Maoqing

**Affiliations:** Department of Drama, Zhejiang Conservatory of Music, Hangzhou, Zhejiang, China

**Keywords:** music performance anxiety, vocal music students, music self-worth, music self-efficacy, music intrinsic motivation

## Abstract

**Introduction:**

This study examines how social support from parents, educators, and peers influences psychological factors—self-efficacy, self-worth, and intrinsic motivation—and how these, in turn, affect music performance anxiety (MPA) among vocal music students. It aims to identify both direct and indirect pathways through which social and psychological variables interact to shape MPA.

**Methods:**

A total of 526 vocal music students from China participated in this study, with a balanced gender distribution (51.3% male, 48.7% female) and educational levels (55.3% undergraduates, 44.7% postgraduates), ranging in age from 20 to 35 years. Structural Equation Modeling (SEM) was used to test the hypothesised model, incorporating multi-group analysis to explore gender-based differences in pathways.

**Results:**

Findings confirmed that self-efficacy, self-worth, and intrinsic motivation significantly mediated the relationship between social support and MPA. Gender differences emerged in the strength and significance of these pathways, with female students showing stronger indirect effects, particularly through self-worth and intrinsic motivation.

**Discussion:**

This study validates the theoretical model, highlighting the importance of both social and psychological resources in managing performance anxiety. While the mediating role of self-efficacy aligns with existing research, the inclusion of self-worth and intrinsic motivation provides new insights into gender-sensitive dynamics of MPA. These findings offer practical implications for educators and policymakers in designing gender-responsive support strategies for vocal music students.

## 1 Introduction

Performance anxiety is a psychological condition characterized by considerable distress and apprehension in situations requiring performance or evaluation, including examinations, public speaking, athletic competitions, or artistic endeavors such as playing an instrument or acting (Kaleńska-Rodzaj, 2023). This type of anxiety has the following harmful reactions that affect bodily organs in different ways. Many body systems are affected by this form of anxiety, such as musculoskeletal manifestations, motor signs (e.g., headaches or tension), psychological, cognitive (e.g., panic attacks) and behavioral symptoms. People with performance anxiety may use words to describe symptoms such as tachycardia, hyperventilation, sweating, paraesthesia of the hands and feet, and sudden chills in the whole body (Gómez-López and Sánchez-Cabrero, 2023). Cortisol can cause a series of physical symptoms in the body; cortisol secretion often rises during this time, leading to increased muscular tension and pain (Guyon et al., 2020). Psychologically, it has crushing effects, sudden rushes of emotions like discomfort, panic, and overwhelming terror flooding our minds that make the thought of performing feel impossible. Cognitive symptoms add a layer of complexity, as those affected may have trouble with attention and memory, making it impossible to concentrate on work or remember needed information: exam material, speeches, lyrics, and rehearsal scores. The mental breaks lead to performance drops and cycle back into more anxiety (MacAfee and Comeau, 2020; Sims and Ryan, 2023). In addition, performance anxiety can also cause physical symptoms such as tense muscles, shaky hands, and, in more serious cases, nausea, vomiting, or diarrhoea (Butković et al., 2022). Such interconnected symptoms exhibit the complex and multi-layered nature of performance anxiety, directly influencing an individual’s ability to execute well and maintain self-confidence in stressful situations.

In addition, individuals with more severe music performance anxiety may begin to experience distressing physiological symptoms—such as muscle tension, shaking, nausea, or dizziness—well before the performance or even immediately after learning that they are expected to perform (Gómez-López and Sánchez-Cabrero, 2023). The fear of performing well begins much earlier and can have a significant impact on everyday life; this is called anticipatory anxiety. Students who focus on vocal performance are particularly vulnerable to performance anxiety since the expectation for faultless presentation is high (Juncos et al., 2017). Vocal performance career students are particularly susceptible to the adverse effects of performance anxiety in areas related to professional growth than other disciplines (Brunkan, 2018). According to the research, more than 20% of vocal performance students experience different levels of performance anxiety, indicating this type of disorder is quite prevalent in this population (Wang and Yang, 2024). Those who display higher levels of performance anxiety tend to experience effects that go beyond the pre-performance and performance phases; students often report a range of secondary problems, such as allergy anxiety, depressive symptoms, severe sleep disturbance, and even migraine (Wang and Yang, 2024). Those symptoms affect their functioning and overall well-being, making it much harder for them to maintain a healthy lifestyle (Butković et al., 2022; Gómez-López and Sánchez-Cabrero, 2023). The breeding of these myriad physical, psychological and emotional aspects of stress makes it evident that tailored interventions are needed to help students cope with performance anxiety, specifically in domains where the occupational outcomes rely heavily on the ability to perform quickly in public.

The influence of gender on music performance anxiety (MPA) among students majoring in vocal music is substantial, as indicated by the fact that female students have higher MPA levels than their male counterparts, perennially reported in research (Coşkun-Şentürk and Çırakoğlu, 2018). This gap may be attributed to several social, psychological, and cultural factors, such as societal pressure, loss of self-identity and even coping mechanisms (Coşkun-Şentürk and Çırakoğlu, 2018). In performance situations, female singers may feel especially insecure and more vulnerable to evaluation, where they are judged on their appearance and musicianship together (Butković et al., 2022; Ma, 2023). Additionally, women’s tendency to internalize stress and the higher trait anxiety levels present in females also contribute to the increased MPA observed among female undergraduates (Sokoli et al., 2022). While male singers do experience performance anxiety, they frequently display different anxiety patterns compared to female vocalists, as gender-specific socialization may influence their development of fluency and risk-taking skills (Sokoli et al., 2022). Understanding these gender differences in MPA can help tailor strategies for anxiety management and lead to more effective interventions for male and female vocal music students by educators and mental health professionals.

## 2 Literature review

### 2.1 The role of self-efficacy in managing music performance anxiety

Self-efficacy refers to the belief that one can properly perform and succeed at various music-related tasks (e.g., playing an instrument, singing, composing and performing). The idea is a specific manifestation of the broader psychological concept of self-efficacy, conceptualized initially by psychologist Bandura (2001) as confidence to perform behaviors required in specified situations. Self-efficacy, or the belief of a musician about their ability to perform and achieve musical goals successfully, is an essential factor in the experience of performance anxiety (Chen, 2024). This belief is constructed through experiences, whether good performances based on solid practice or experiences where competence is felt.

Self-efficacy is a key factor that influences experiences of vocal music students with strong effects on their levels of MPA (González et al., 2018; MacAfee and Comeau, 2020; Wang and Yang, 2024). It is a singer’s belief in their ability to carry out vocal tasks and face musical challenges, from navigating complex pieces, controlling the voice, and performing with feeling. Self-efficacy has been shown to encourage learners to approach performances with higher confidence, maintain greater effort during practice, and stick with challenges so that overall performance quality improves (McCormick and McPHERSON, 2003; Stothert, 2012). This confidence acts as a buffer against the nerves and anxiety of performance by allowing students to feel prepared going into performances, less like impostors and more like they know what they are doing, thereby reducing potential anxiety, fear or self-doubt that might occur leading up to or in the middle of a performance (Henshaw and Collyer, 2022). On the other hand, reduced self-efficacy may exacerbate MPA, driving students to doubt their singing abilities, fear negative evaluations, which leads to amplified anxiety and somatic responses as well as possible avoidance mechanisms. Previous achievements or failures in performances, the quality of practice sessions, and feedback from educators, peers and audiences shape the development of self-efficacy in vocal music (Moore, 2024). When a student effectively receives positive feedback or encouragement, it generally boosts the self-efficacy of that student; on the contrary, consistent unpleasant experience or severe criticism reduces the level of self-belief, due to which he/she becomes more prone to worry. Hence, a strong self-efficacy is one of the foundational stones for students who study vocal music, as it reduces the fear of performance and brings a positive attitude towards their work.

### 2.2 Self-worth and its psychological impact on vocal performance

Self-worth is the overall sense of value one has in themselves and believing that one is worthy of respect and acceptance, regardless of specific accomplishments and outside validation (Detmer et al., 2020). It encapsulates the notion of oneself in terms of personal worth, often shaped by what people have been through or influenced by social comparisons and internal standards. People who have a balanced and positive self-esteem approach challenges with an optimal mindset, since their success or failure has little impact on their sense of worth (Harpaz and Vaizman, 2023). When self-worth is predominantly linked to external elements such as achievements, validation from others, or social standing, it becomes more precarious and vulnerable to variations influenced by perceived success or failure across different aspects of life. This tenuous self-worth may cause individuals to anchor their self-esteem to particular results, potentially resulting in increased sensitivity to criticism, fear of failure, and the evasion of circumstances where their competencies could be assessed (Vispoel, 2021).

In the realm of music performance, self-worth is frequently associated with a musician’s assessment of their abilities, accomplishments, and the acknowledgement they obtain from audiences, colleagues, and mentors (Sickert et al., 2022). Musicians, particularly students in training, may conflate their self-worth with their musical ability, leading them to believe that their personal value is contingent upon the quality of their performances. When self-worth hinges on musical achievement, performance anxiety tends to escalate due to the heightened perceived stakes; a subpar performance is regarded not merely as a competence deficiency but as an indication of personal weakness (Antonini Philippe et al., 2022). The apprehension of poor evaluation or inability to fulfil expectations can exacerbate anxiety symptoms, including tension, self-doubt, and physical stress, further hindering performance quality (Antonini Philippe et al., 2022). In contrast, artists with a stable sense of self-worth independent of their musical accomplishments may have diminished performance anxiety, as they exhibit greater resilience to failures and are less inclined to associate performance outcomes with their intrinsic value.

### 2.3 Intrinsic motivation as a buffer against performance-related anxiety

Intrinsic motivation refers to the drive to engage in activities for the inherent enjoyment, satisfaction, or challenge value of doing so instead of for external rewards or pressures (Woody, 2021). Intrinsically motivated people do things for their own sake for the sense of satisfaction from doing them, so they are more devoted and engaged. Because the know-how is excited about their work and motivated by intrinsic motivation, this motivation frequently ends in elevated sustained interest and perseverance, as people engage in projects driven by genuine interest and curiosity (Wang and Wong, 2022). On the other hand, if on most occasions motivation is more often than not extrinsically driven by outside incentives such as rewards, recognition or running from punishment, the attention is completely diverted away from the intrinsic value of doing something. That change may reduce the fun and disrupt the long-term rewards from doing the task, because now the primary motivation is driven by an outcome rather than self-satisfaction or interest (López-Íñiguez et al., 2022). Encouraging intrinsic motivation is a key factor to mastering something and maintaining mental health, as it leads people to focus on the learning and development process rather than just the outcome.

Intrinsic motivation plays a vital role in determining the experiences and emotional responses of a musician in the particular field of music performance (López-Íñiguez et al., 2022). With their intrinsic motivation, music students could participate in practice and performances. The work contains rewards, such as joy in singing or playing an instrument, a deep connection with the music, and personal expression through art. Coming out of such intrinsic motivation, resilience increases. Stress decreases and the quality of performance punches way above its weight. Such intrinsic drive can further strengthen resilience, reduce stress and improve performance quality; students are focused on their music-driven passion instead of any external pressure. Self-worth might be profoundly influenced by intrinsic drive and MPA (Hu, 2024). When music students associate their self-worth with their musical accomplishments, they may become excessively preoccupied with seeking external validation, securing victories in contests, or evading failure. This redirects their drive towards external considerations, perhaps increasing performance anxiety as they perceive their worth is jeopardized with each performance. When self-worth is steady and independent of musical accomplishment, students are more inclined to sustain intrinsic motivation and engage with music through curiosity and enjoyment, resulting in reduced anxiety levels (Papageorgi, 2022). Consequently, cultivating a robust sense of intrinsic motivation in conjunction with a stable self-worth is crucial for assisting music students in managing performance anxiety and achieving fulfilment in their musical endeavors.

### 2.4 Social support from parents, educators, and peers in vocal music education

Parental support is essential in determining how a music student addresses and controls performance anxiety, significantly affecting their emotional resilience and attitude to musical performances (Oliveira et al., 2024). Also, the emotional climate parents create may significantly shape youth attitudes toward public performances, a process that is especially personal and involves much vulnerability (Tardif et al., 2024). When parents create a supportive, nurturing, and understanding environment at home, it helps build students’ self-esteem and reduces their fear of rejection or failure (Barnes et al., 2016). Because singers and instrumentalists often face frequent evaluations, their confidence can easily fluctuate, making emotional support an essential part of helping them cope with the pressures of music performance (Kong, 2023). Parents who praise their child for effort, evolution, and improvement instead of performance results could be part of an integral step in developing a growth mindset. Such an approach promotes a greater appreciation for the process of learning and a focus on effort as well as mastery, rather than perfection or external success, leading to a healthier and more sustainable model for musical growth (Kenny and Holmes, 2018). This shifts the attention away from fear of failing and towards a more positive view of skill development, thus reducing performance anxiety. Moreover, parents also model healthy coping strategies; by responding calmly and constructively to stress and challenges, they provide children with crucial tools for managing anxiety (Huang and Yu, 2022). Parents who show persistence and a positive perspective in the face of failure teach their children helpful skills to deal with performance anxiety and negative feelings (Tardif et al., 2024). Such a broad support structure not only facilitates the often overwhelming aspects of music extemporizing but also cultivates an enduring regard for an art form as a vehicle for self-growth and enjoyment.

The pedagogical decisions, feedback strategies and classroom culture established by educators play an important role in students’ experience with musical performance anxiety, as these characteristics of the learning/teaching environment can alleviate or exacerbate levels of anxiety experienced (Stijnen et al., 2024). Educators successfully create a strong base of confidence in students by employing a student-centered approach focused on developing skills and people. Constructive and helpful feedback will encourage students to focus on their growth trajectory or strengths over their weaknesses, which can greatly lower performance anxiety (Mazzarolo et al., 2023). Creating a safe, non-judgmental learning environment where students feel valued and understood helps to build their confidence so that when it comes time to perform in front of an audience, they will be more resilient against the pressures. According to de Bruin (2023), carefully implemented tasks, such as simulated performances that mimic real performance settings, relaxation and breathing exercises to calm the mind and body, and the use of imagery for positive visualization to promote a confident mental state before performing, can proactively reduce performance anxiety disturbances in classroom environments. Integrating such activities into regular classes allows educators to develop students’ musicality and psychological and emotional toolkit for handling performance pressure. Then, educators build community in their classrooms by encouraging open conversations about anxiety and helping students know they are not alone with their worries. Creating an environment where students can share and discuss their concerns builds a sense of community, making the learning context a safe space to address performance anxiety and build confidence through each other’s experiences (Jääskeläinen et al., 2023). This comprehensive approach complements the musical technicalities, equipping students to face performance issues by charging their emotional reserve capacity and a positive mindset.

Peer support is significant for vocal music students because of the potential for anxiety related to performance, and this often leads to relieving tension and reducing feelings of isolation through peer interaction that entails both encouragement and empathy. Articulating that particular experience of fighting against the challenges involved in performing a music piece develops a bond with peers, which is key to coping with performance anxiety (Bussu and Mangiarulo, 2024; Sims and Ryan, 2023). If students see their friends facing similar hurdles, then it evokes a bond in the fight against fear; it normalizes the fear and makes them less likely to think of it as a shortcoming on their part. Social support can come in the form of simply encouragement, sharing coping skills that work for them or just listening and offering sympathy when times are tough (Vengurlekar et al., 2024). This is attractive because counsel and comfort from peers are often more user-friendly and less intimidating than guidance from professors or authority figures, making it easier for students to take in or avail of the support they provide (Tahirbegi, 2023). In addition, peer-oriented activities like group rehearsals and ensembles can help students prepare for performance in a less formal, more collaborative setting for an audience (Tahirbegi, 2023). Having this opportunity in a safe environment could help gently build students’ confidence and acclimatize them to the anxiety associated with performing publicly. By participating in these cooperative experiences, students develop their performance skills and learn how to cope with the emotional distress that often accompanies performing while learning to reduce performance anxiety over time through availing resilience and support from each other.

### 2.5 Theoretical frameworks underpinning the study

This study draws upon three complementary theoretical frameworks to construct and interpret the proposed model of music performance anxiety (MPA): the Stimulus–Organism–Response (SOR) theory (Mehrabian and Russell, 1974), Social Cognitive Theory (SCT) (Bandura, 1978), and Self-Determination Theory (SDT) (Ryan and Deci, 2000). The SOR framework explains how external stimuli (e.g., support from parents, teachers, and peers) influence internal psychological states (e.g., self-worth, motivation, and efficacy), subsequently affecting behavioral responses such as MPA. This structure is the foundation for conceptualizing the model’s interaction between social and psychological factors. In addition to the SOR framework, SCT focuses on self-efficacy and observational learning, elucidating how students’ beliefs in their performance abilities—shaped by social modelling and prior experiences—impact motivation and anxiety in music contexts. SDT emphasizes the importance of three fundamental psychological needs—autonomy, competence, and relatedness—in promoting intrinsic motivation. In music education, satisfying these needs through supportive social environments can reduce performance-related anxiety and promote confident engagement.

### 2.6 This study

The primary aim of this study is to examine how different sources of social support, specifically from parents, educators, and peers, influence key psychological constructs, including self-efficacy, self-worth, and intrinsic motivation, and how these constructs affect MPA among vocal music students. Drawing on the SOR framework, SDT, and SCT, this study also explores whether gender moderates these relationships and whether psychological factors mediate the effects of social support on MPA. The study seeks to develop and validate a structural model that comprehensively understands the psychological pathways leading to performance anxiety in music education contexts.

#### 2.6.1 Research framework

This study frames support from parents, educators, and peers as stimuli that attract students’ experience with mediating factors such as self-efficacy, self-worth, and intrinsic motivation, which are the properties of an organism. In terms of this framework, the response is MPA, which demonstrates how these internal and external influences interact, influencing vocal students’ anxiety levels. Moreover, it answers students’ concerns regarding MPA while placing additional context on our understanding of how gender differences may shape individuals’ experiences. Analyzing these relationships will help shed light on the role of various types of social support as well as automatic thoughts in shaping performance anxiety among vocal students.

**Figure 1:**
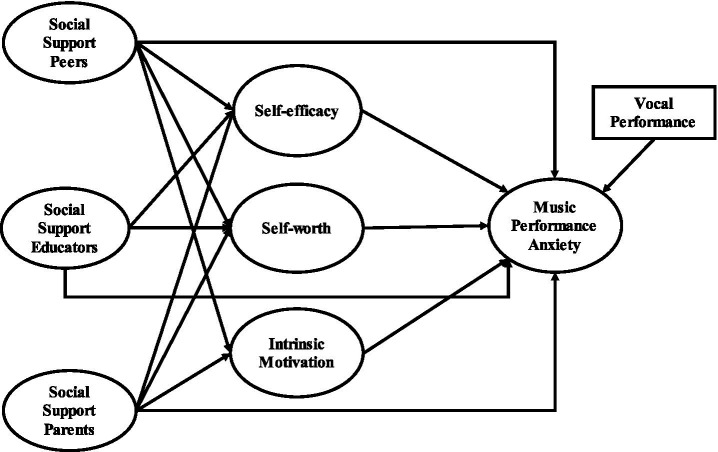
Research model.

The proposed model is illustrated in Figure 1.

#### 2.6.2 Research questions

RQ1: How does social support from parents, educators, and peers relate to self-efficacy, self-worth, and intrinsic motivation among vocal music students, and do these relationships differ by gender?

RQ2: What is the relationship between self-efficacy, self-worth, intrinsic motivation, and music performance anxiety, and how does gender moderate these associations?

RQ3: Do self-efficacy, self-worth, and intrinsic motivation mediate the relationship between social support (parents, educators, and peers) and music performance anxiety in vocal music students?

#### 2.6.3 Research hypotheses

The present study proposes a structured set of hypotheses grounded in a confirmatory framework to explore how social support from parents, educators, and peers influences MPA in vocal music students through three key psychological mediators: self-efficacy, self-worth, and intrinsic motivation. Specifically, it is hypothesized that social support from parents (H1a), educators (H1b), and peers (H1c) will positively predict self-efficacy (H1), while the same sources are also expected to positively predict self-worth (H2a, H2b, H2c) and intrinsic motivation (H3a, H3b, H3c), respectively, representing H2 and H3. With respect to MPA, it is proposed that higher levels of social support from parents (H4a), educators (H4b), and peers (H4c) will negatively predict anxiety levels (H4), and that higher self-efficacy (H4d), self-worth (H4e), and intrinsic motivation (H4f) will each independently reduce MPA, indicating their protective roles. Vocal performance is also expected to directly predict lower MPA (H4g). Furthermore, the model includes mediation hypotheses positing that self-efficacy mediates the effect of parental, educator, and peer support on MPA (H5a, H5b, H5c); self-worth mediates these same relationships (H6a, H6b, H6c); and intrinsic motivation serves as a third mediator (H7a, H7b, H7c). These hypotheses collectively aim to identify the direct and indirect mechanisms by which social and psychological variables interact to influence MPA in the context of vocal performance (see Tables 1, 2).

**Table 1:** Direct effect.

Hypotheses	Men		Women	
	Estimate	*p*-value	Estimate	*p*-value
H1: Self-efficacy				
H1a: Social support parents → Self-efficacy	0.339	<0.001	0.611	<0.001
H1b: Social support educators → Self-efficacy	0.611	<0.001	0.409	<0.001
H1c: Social support peers → Self-efficacy	0.065	0.102	0.396	<0.001
H2: Self-worth				
H2a: Social support parents → Self-worth	0.378	<0.001	0.563	<0.001
H2b: Social support educators → Self-worth	0.653	<0.001	0.437	<0.001
H2c: Social support peers → Self-worth	0.098	0.134	0.439	<0.001
H3: Intrinsic motivation				
H3a: Social support parents → Intrinsic motivation	0.109	0.093	0.529	<0.001
H3b: Social support educators → Intrinsic motivation	0.719	<0.001	0.521	<0.001
H3c: Social support peers → Intrinsic motivation	0.298	0.019	0.309	<0.001
H4: MPA				
H4a: Social support parents → MPA	−0.498	<0.001	−0.309	<0.001
H4b: Social support educators → MPA	−0.618	<0.001	−0.346	<0.001
H4c: Social support peers → MPA	−0.105	0.109	−0.568	<0.001
H4d: Self-efficacy → MPA	−0.383	<0.001	−0.662	<0.001
H4e: Self-worth → MPA	−0.568	<0.001	−0.621	<0.001
H4f: Intrinsic motivation → MPA	−0.266	0.026	−0.586	<0.001
H4g: Vocal performance → MPA	−0.439	<0.001	−0.388	<0.001

**Table 2:** Indirect effect.

Indirect hypotheses	Men		Women	
	Estimate	*p*-value	Estimate	*p*-value
H5a: Social support parents → Self-efficacy → MPA	−0.130	0.078	−0.404	<0.001
H5b: Social support educators → Self-efficacy → MPA	−0.234	0.021	−0.271	<0.001
H5c: Social support peers → Self-efficacy → MPA	−0.025	0.289	−0.262	0.016
H6a: Social support parents → Self-worth → MPA	−0.215	0.033	−0.350	<0.001
H6b: Social support educators → Self-worth → MPA	−0.371	<0.001	−0.271	<0.001
H6c: Social support peers → Self-worth → MPA	−0.056	0.209	−0.273	<0.001
H7a: Social support parents → Intrinsic motivation → MPA	−0.029	0.254	−0.310	<0.001
H7b: Social Support Educators → Intrinsic motivation → MPA	−0.191	0.044	−0.305	<0.001
H7c: Social Support Peers → Intrinsic motivation → MPA	−0.079	0.109	−0.181	0.058

## 3 Materials and methods

### 3.1 Measures of research variables

The current study examines seven latent variables (i.e., self-efficacy, self-worth, intrinsic motivation, parental support, educator support and peer support and MPA) and two measurable (gender and vocal performance). Each latent variable used a 7-point Likert scale, wherein participants indicated how strongly they agreed or disagreed with statements about the constructs. Respondents rated each item on a scale of 1–7, with a score of 1 indicating strong disagreement and 7 indicating strong agreement. Participants were asked to rate their perspectives and experiences regarding the support received from parents, educators, and peers, as well as their self-efficacy, self-worth, and intrinsic motivation. The MPA levels were assessed through a scale they developed which takes into consideration the severity of anxiety experienced within musical activities. In addition, gender was treated as a categorical variable (male or female) to examine potential moderation effects. Vocal performance experience was assessed through participants’ self-reported number of years engaged in formal vocal training or performance.

#### 3.1.1 Social support (parents, educators, and peers)

The Social Support Scale, Ryan et al. (2000) is a psychological tool designed to evaluate perceived social support in a variety of life situations. Based on the SDT, this instrument denotes that social support can foster psychological development, intrinsic motivation and general wellbeing. There is for example assessment of what people think of their social environment that emphasize to what extent they believe interpersonal exchanges are supportive, caring and autonomy conducive (Zarza-Alzugaray et al., 2020). The essential idea is that social support is much more than the simple appearance of other people; it refers to the quality of those interactions and ability to satisfy basic psychological needs such as autonomy, competence and relatedness (Zarza-Alzugaray et al., 2020).

The Social Support Scale consists of several questions or statements that the respondents respond to and reflect their perceived levels of support in different types of relationships, such as those with parents, educators and peers. It includes various aspects of social support such as emotional support (extent to which persons perceive care and worth); instrumental support (provision of tangible assistance); and informational support (availability of advice and guidance). The scale also includes an autonomy support dimension, which reflects how much of the social environment is supportive of independent decision making and pursuit of their own interests. The Social Support Scale has been widely used in psychology studies (Huawei and Jenatabadi, 2024; Orejudo et al., 2021), and it examine the relationship between social support with several constructs such as mental health, motivation, well-being etc. The emphasis on autonomy support makes it especially useful in settings where cultivating self-determination and personal development is essential, including schools, workplaces, or therapeutical environments. It assesses to what extent social networks promote independence and self-determination of personal development in context, providing a scale that some thought may contribute more effectively towards overall well-being with the help of social support.

All items were measured using a 7-point Likert scale ranging from 1 (strongly disagree) to 7 (strongly agree).

Example items:

- *I feel emotionally supported by my parents when I perform.*
- *My educators help me overcome anxiety before performances.*
- *My classmates encourage me to succeed.*

#### 3.1.2 Self-efficacy

To assess the sources contributing to participants’ vocal self-efficacy, this study employed an adapted version of Zelenak (2011) Music Performance Self-Efficacy Scale (MPSES). The original version of the MPSES consisted of 24 items to evaluate four main sources pertaining to self-efficacy: mastery experience (direct personal accomplishments in doing), verbal persuasion (encouragement and feedback by others), vicarious experience (observation on performances done by others) and physiological status (personal interpretation regarding its physical condition, with reference to the effect from positive or negative related cues in performing). For the present study, the scale was adapted with minor modifications to ensure that all items were appropriately contextualized for vocal performance, while preserving the theoretical integrity of Zelenak’s (2011) original framework. These adaptations included rewording the items in relation to their experience and perception of singing and vocal skills. The revised scale was used to measure the extent to which each of the four sources influenced the participants’ vocal self-efficacy, offering a more targeted evaluation of the factors shaping their confidence in vocal performance. Using this method offered the opportunity to deeply delve into the individual factors of vocal self-efficacy, offering thematically driven and nuanced accounts of how particular experiences or responses lead to either building or diminishing confidence in singing.

All items were measured using a 7-point Likert scale ranging from 1 (strongly disagree) to 7 (strongly agree).

Example items:

- *Seeing classmates succeed boosts my confidence.*
- *Encouraging comments make me feel capable.*
- *Physical signs help me understand my readiness.*

#### 3.1.3 Self-worth

Adapting content from Xu (2023) self-worth theory, this study assesses self-worth with 11 items that are specifically developed. Early studies by Covington showed that self-worth is closely related to a person’s perceived ability to achieve or succeed in situations that require performance. To make this construct applicable for the current research, items have been developed to better capture individual differences pertaining specifically to vocal performance. These items aim to measure participants’ valuation of themselves based on their singing ability and accomplishments, thereby allowing the assessment to reflect one core dimension of self-worth that may influence experiences of MPA.

All items were measured using a 7-point Likert scale ranging from 1 (strongly disagree) to 7 (strongly agree).

Example items:

- *I respect myself even when I make mistakes.*
- *My self-worth does not depend on others’ approval.*
- *I have confidence in my personal abilities.*

#### 3.1.4 Intrinsic motivation

The present study used the interest/enjoyment subscale from Ryan’s (1982). The Intrinsic Motivation Inventory (IMI), with appropriate modifications, assesses participants’ intrinsic motivation to sing using their voice. The IMI is a widely established instrument for evaluating multiple dimensions of intrinsic motivation, with the interest/enjoyment dimension recognized as the most direct indicator of intrinsic motivation. This subscale measures the extent to which an activity is pursued for personal enjoyment, inherent satisfaction, and interest rather than for external pressures or rewards.

In this study, all items were explicitly adapted to the context of vocal music. Each item was rephrased to reflect singers’ authentic experiences, perceptions, and emotional responses during vocal practice and performance. These modifications ensure that the instrument accurately captures the nuances of intrinsic motivation in vocal performance settings while preserving the theoretical integrity of the original scale. By focusing on enjoyment, interest, and self-driven engagement, this tailored version of the IMI offers a precise understanding of how intrinsic motivation influences vocal music students’ commitment and satisfaction in their singing activities.

To illustrate, sample items from the adapted scale include:

- *I sing because I genuinely enjoy the process.*
- *Practicing vocal music is fun and engaging for me.*
- *I feel excited and motivated whenever I have the opportunity to sing.*

All items were measured using a 7-point Likert scale, ranging from 1 = strongly disagree to 7 = strongly agree, allowing participants to express the degree to which each statement reflected their personal experience.

#### 3.1.5 MPA

Vocal music students must deal with specific anxiety when performing vocal pieces. Thus, we would still need to develop tools precisely for measuring MPA, as little research is focused on this area. To fill that void, the current study employs and adapts components of three pioneering research studies Kenny (2009), Papageorgi et al. (2007), and Ryan and Andrews (2009) in an effort to develop a measurement scale specific to students who were enrolled in vocal music courses. These works offer broad tools for surveying aspects of MPA, theoretical models to explain the causes and manifestations of the condition, and specific details regarding experiences from singers that include but are not limited to vocal control and fear of judgment. Thereby, incorporating knowledge from these sources, the present research created a 14-item scale to reflect vocal music students’ distinctive issues and ailments. The scales cover several factors of MPA (physical symptoms, cognitive fears, emotional reactions, and social influences); however, the specific items relate to the singer’s experience, including voice break fear (i.e., worry that your voice will break when you sing) (Yeakel et al., 2024) and perceived vocal strain (Stone and Erickson, 2024). This personalized treatment seeks to give a more valid and complete source for measuring MPA in voice students, publishing findings that provide critical information lacking in the literature on this topic.

All items were measured using a 7-point Likert scale ranging from 1 (strongly disagree) to 7 (strongly agree).

Example items:

- *My hands tremble when I sing in front of others.*
- *My heart races before I go on stage.*
- *My anxiety affects my vocal control.*

Table 3 provides an overview of the theoretical foundations supporting each measurement variable employed in this study. Additionally, the Supplementary file includes a comprehensive list of all latent variables and their corresponding questionnaire items.

**Table 3:** Theoretical support for measurement variables.

Latent variable	Dimensions	Quantity of inquiries	Theoretical support
Social support parents	–	5 items	Ryan et al. (2000)
Social support educator	–	5 items	Ryan et al. (2000)
Social support peers	–	5 items	Ryan et al. (2000)
Self-efficacy		24 items	Zelenak (2011)
	Enactive Mastery Experience [6 items]		
	Vicarious Experience [6 items]		
	Verbal/Social Persuasion [6 items]		
	Physiological and Affective State [6 items]		
Self-worth	–	11 items	Xu (2023)
Intrinsic Motivation		24 items	Ryan (1982)
	Interest [4 items]		
	Perceived Competence [4 items]		
	Importance [4 items]		
	Tension [4 items]		
	Perceived Choice [4 items]		
	Value [4 items]		
	Interest [4 items]		
MPA	–	14 items	Kenny (2009), Papageorgi et al. (2007), and Ryan and Andrews (2009)

### 3.2 Data collection process

Power analysis using G*Power, version 3.1.9, suggested a minimum sample size of 468 participants was necessary for this study to achieve the desired statistical power. This computation was based on an assumed effect size of 0.15, alpha = 0.05 and power = 0.95, ensuring a high likelihood of detecting meaningful effects in the current study. The survey questions were first developed in the English language and were then carefully reviewed by two experts who are fluent in both Chinese and English. A back-translation method was employed to guarantee accuracy: the original instrument was translated from English into Chinese and thereafter retranslated from Chinese into English to keep both consecutive language versions identical and clear.

The present study was conducted at 24 universities throughout China that offer undergraduate or graduate degrees in music education. We contacted each university before the proposed institutional collaboration, through formal communication channels, specifically addressing heads of departments, deans of research, and deputy deans of research. We explained the research project, including its aims and objectives and significance, and that the study had been granted full ethical approval from an academic review board. Furthermore, we have emphasized that all ethical requirements and regulations were strictly adhered to, in accordance with national and institutional research ethics guidelines. Written informed consent was obtained from all participants before their inclusion in the study. The Zhejiang Conservatory of Music Ethics Committee approved this research ethically (Reference: 2024-ZYKY-01; Date: July 10, 2024).

Of the 24 contacted universities, 18 universities were willing to cooperate and actively contributed with relation to the study, such as accessibility to the collection of data. Data were collected through an online questionnaire distributed in WeChat groups, a widely used platform for academic exchanges and negotiations among music students. These groups were both undergraduate and graduate vocal music students and thus represented a more diverse selection.

Before proceeding, all respondents were presented with a full informed consent sheet in the first page of the survey, which explained the objectives of the research, the voluntary nature of their participation, and guarantees of anonymity and confidentiality in the responses. Only respondents who answered “yes, I agree to participate” could advance to the main questionnaire.

The study was done in accordance with ethical standards for research. Participants were told they could stop anytime without any reason or cost.

#### 3.2.1 Inclusion and exclusion criteria

Eligible participants were undergraduate or postgraduate students enrolled in a music program at the time of data collection, majoring in vocal performance or a vocal-related music education field, aged 20 years or older, and capable of providing informed consent for participation in the study.

Exclusion criteria involved: Nonvocal music major students (e.g., instrumentalists, composers, musicology students), Students under 20 years of age, Incomplete questionnaires or answers that did not pass quality control checks (e.g., patterned answers; paucity of answers), and individuals who lacked informed consent.

### 3.3 SEM process

#### 3.3.1 Validity, reliability, and multicollinearity analysis

To assess the quality of the measurement model, this study evaluated construct reliability, convergent validity, and multicollinearity following guidelines by Fornell and Larcker (1981) and Hair et al. (2019). Reliability was tested using Cronbach’s Alpha, while convergent validity was determined by calculating the Average Variance Extracted (AVE). Discriminant validity was supported when each construct’s AVE exceeded 0.50. To ensure no severe multicollinearity, Variance Inflation Factor (VIF) values were also examined, with thresholds below 5 considered acceptable (Hair et al., 2019).

Table 4 presents the psychometric properties of the latent variables used in this study. All constructs showed acceptable internal consistency, with Cronbach’s alpha values above the minimum threshold of 0.70. The AVE values for all constructs exceeded the 0.50 benchmark, confirming convergent validity. VIF values for all observed items ranged between 1.88 and 3.92, indicating that multicollinearity was not a concern in this model. These results suggest that the model constructs are both reliable and valid for SEM.

**Table 4:** Validity, reliability, and multicollinearity analysis.

Construct	Cronbach’s alpha	AVE	VIF range
Parental support	0.751	0.564	2.44–3.85
Educator support	0.752	0.582	2.28–3.80
Peer support	0.862	0.598	2.59–3.77
Self-efficacy	0.793	0.584	2.11–3.54
Self-worth	0.886	0.551	1.98–2.98
Intrinsic motivation	0.778	0.619	2.15–3.21
Emotional intelligence	0.759	0.566	2.27–3.37
MPA	0.824	0.601	2.38–3.93

#### 3.3.2 Model fitting

The overall model fit was evaluated using multiple fit indices: Chi-square (χ^2^), Comparative Fit Index (CFI), Tucker-Lewis Index (TLI), Root Mean Square Error of Approximation (RMSEA), Standardized Root Mean Square Residual (SRMR), Goodness of Fit Index (GFI), and Adjusted Goodness of Fit Index (AGFI). Table 5 presents the model fitting results for the full structural equation model, showing that all key fit indices meet or exceed widely accepted benchmarks for SEM.

**Table 5:** Model fitting.

Fit index	Threshold	Full SEM	Interpretation
Chi-square (χ^2^)	Non-significant (ideal)	847.35	Acceptable with large N
CFI (Comparative Fit Index)	≥0.90–0.95	0.928	Good fit
TLI (Tucker-Lewis Index)	≥0.90	0.917	Good fit
RMSEA (Root Mean Square Error of Approximation)	<0.08	0.061	Good fit
SRMR (Standardized Root Mean Residual)	<0.08	0.049	Good fit
GFI (Goodness of Fit Index)	≥0.90	0.901	Acceptable fit
AGFI (Adjusted GFI)	≥0.85	0.883	Acceptable fit

#### 3.3.3 Model estimation

SEM was conducted using AMOS 24.0 with Maximum Likelihood Estimation (MLE). To ensure the robustness of the hypothesized model and evaluate the structural relationships in a theoretically grounded and stepwise manner, we conducted three Confirmatory Factor Analyses (CFAs) on conceptually grouped sub-models derived from the proposed framework. This approach allowed us to assess individual components’ construct validity and measurement adequacy before integrating them into the full SEM model. These sub-models reflect the pathways from social support to psychological mediators (self-efficacy, self-worth, and intrinsic motivation) and their relationship with MPA.

All three sub-models demonstrated an acceptable to good fit across conventional SEM indices. Model 1 and Model 3 showed strong alignment with theoretical expectations, with no need for post-hoc modifications. Model 2 required minimal adjustment to account for correlated errors between peer support items, a change theoretically supported due to item content overlap. These results confirm that each pathway linking social support to psychological mediators and MPA was well-specified and suitable for integration into the overall structural equation model (see Table 6).

**Table 6:** Confirmatory factor analyses (sub-models).

Sub-model	Variables included	Fit indices	Acceptable thresholds	Model modifications
*Model 1*: Social Support → Self-efficacy → MPA	Parental, Educator, Peer Support; Self-Efficacy; MPA	CFI = 0.93, TLI = 0.91, RMSEA = 0.056, SRMR = 0.049	CFI/TLI ≥ 0.90, RMSEA < 0.08, SRMR < 0.08	None
*Model 2*: Social Support → Self-worth → MPA	Parental, Educator, Peer Support; Self-Worth; MPA	CFI = 0.91, TLI = 0.89, RMSEA = 0.061, SRMR = 0.051	CFI/TLI ≥ 0.90, RMSEA < 0.08, SRMR < 0.08	Covariance added between two peer support indicators
*Model 3*: Social Support → Intrinsic Motivation → MPA	Parental, Educator, Peer Support; Intrinsic Motivation; MPA	CFI = 0.94, TLI = 0.92, RMSEA = 0.053, SRMR = 0.047	CFI/TLI ≥ 0.90, RMSEA < 0.08, SRMR < 0.08	None

## 4 Results

### 4.1 Descriptive and correlation analyses

The study involved 526 vocal music students who participated in a survey. Among these participants, 270 were male (51.3%) and 256 were female (48.7%), indicating a nearly balanced gender distribution. Regarding academic level, 55.3% of the participants were undergraduate students and 44.7% were postgraduate students, suggesting the sample was drawn from various scholastic stages. There was a wide age gap among participants, with the youngest being 20 and the oldest 35. 26.4% of the students were under 22 years old, 32.7% were between 22 and 28, 22.4% were between 29 and 32, and 18.4% were older than 32. The age and gender distribution presented here demonstrates that the current investigation includes early career as well as experienced vocal music students, thus offering a wide view of music performance anxiety and its psychological correlates across gender and age ranges.

Table 7 presents the descriptive statistics for the key variables included in the study, based on responses from 526 vocal music students. On a 7-point Likert scale, students reported moderate to high levels of parental (*M* = 5.31, SD = 1.33), educator (*M* = 5.12, SD = 1.21), and peer support (*M* = 5.37, SD = 1.02). Psychological constructs also demonstrated relatively high means: self-worth (*M* = 4.96), self-efficacy (*M* = 5.63), and intrinsic motivation (*M* = 5.82), suggesting generally positive perceptions. The average music performance anxiety (MPA) score was notably lower (*M* = 3.35, SD = 1.19), indicating moderate anxiety levels in the sample. The observed vocal performance mean was 4.29 (SD = 1.69), while the mean age of participants was 25.41 years (SD = 3.69). These values provide an overview of the sample’s psychological profile and musical context before structural modelling.

**Table 7:** Descriptive statistics of study variables (*N* = 526).

Variable	Mean (M)	Standard deviation (SD)	Scale range
Parental support	5.31	1.33	1–7
Educator support	5.12	1.21	1–7
Peer support	5.37	1.02	1–7
Self-efficacy	5.63	1.29	1–7
Self-worth	4.96	1.35	1–7
Intrinsic motivation	5.82	0.93	1–7
MPA	3.35	1.19	1–7
Vocal performance (Observed)	4.29	1.69	1–7
Age	25.41	3.69	20–35

Table 8 presents the Pearson correlation coefficients among the key latent variables and their dimensions used in the study, based on data from 526 participants. The results reveal moderate to strong positive correlations among different sources of social support (parents, educators, peers) and dimensions of intrinsic motivation (e.g., interest, perceived competence, value), indicating that greater perceived support is associated with higher motivational constructs. Notably, negative correlations are observed between MPA and several predictors such as enactive mastery experience (*r* = −0.52), perceived competence (*r* = −0.42), and self-worth (*r* = −0.49), suggesting that stronger psychological resources and support systems are associated with lower levels of music performance anxiety. Tension and physiological/affective states show positive correlations with MPA and negative correlations with self-efficacy and motivational variables, reinforcing their maladaptive role in anxiety.

**Table 8:** Correlation analysis.

Variable	[1]	[2]	[3]	[4]	[5]	[6]	[7]	[8]	[9]	[10]	[11]	[12]	[13]	[14]	[15]
[1] Social support parents	1														
[2] Social support educator	0.57	1													
[3] Social support peers	0.61	0.65	1												
[4] Interest	0.38	0.45	0.52	1											
[5] Perceived competence	0.41	0.42	0.45	0.58	1										
[6] Importance	0.42	0.51	0.49	0.62	0.56	1									
[7] Tension	−0.3	−0.26	−0.29	−0.4	−0.33	−0.31	1								
[8] Perceived choice	0.36	0.4	0.38	0.55	0.5	0.52	−0.39	1							
[9] Value	0.38	0.41	0.42	0.55	0.51	0.57	−0.37	0.46	1						
[10] Enactive mastery experience	0.45	0.5	0.43	0.48	0.53	0.47	−0.4	0.41	0.46	1					
[11] Vicarious experience	0.36	0.45	0.48	0.41	0.45	0.41	−0.39	0.44	0.43	0.57	1				
[12] Verbal/Social persuasion	0.41	0.39	0.45	0.38	0.42	0.44	−0.37	0.4	0.45	0.53	0.56	1			
[13] Physiological and affective state	−0.39	−0.37	−0.4	−0.45	−0.42	−0.43	0.42	−0.41	−0.44	−0.46	−0.4	−0.4	1		
[14] Self-worth	0.47	0.5	0.52	0.59	0.57	0.56	−0.36	0.55	0.59	0.63	0.55	0.54	−0.45	1	
[15] MPA	−0.31	−0.33	−0.34	−0.45	−0.42	−0.39	0.5	−0.41	−0.44	−0.52	−0.44	−0.44	0.58	−0.49	1

### 4.2 Structural model

The structural model is a fundamental component of SEM, as it delineates the proposed links between latent variables (unobserved constructs) and, in certain instances, the associations between latent and observed variables. This concept is generally represented by a route diagram, which visually conveys the network of interconnections by directed arrows that signify supposed causal connections. The structural model comprises a set of regression-like equations that delineate the interactions of latent variables and their influence on observable variables, grounded in theoretical assumptions. The arrows linking the variables denote paths, with coefficients allocated to these pathways that measure the strength and direction of the associations, indicating whether the influence is positive or negative and its magnitude. The structural model facilitates the examination of complex theoretical frameworks by modelling connections, thereby elucidating the intricate interactions among many components and offering insights into the mechanisms underlying observable data patterns. Table 1 presents the direct effects among the study variables, while Table 2 complements these findings by reporting the indirect (mediated) effects, together offering a comprehensive understanding of how social support influences MPA both through immediate pathways and through psychological mediators.

The analysis reveals substantial correlations among social support, self-efficacy, self-worth, intrinsic motivation, and MPA for both genders, with distinct gender differences in the intensity of these interactions. Social support from parents and educators is a significant predictor of self-worth for both genders (*p* < 0.001), with a higher influence from educators on men (0.653) and from parents on women (0.563). Peer support significantly influences women’ self-worth (0.439, *p* < 0.001), whereas it does not affect men (0.098, *p* = 0.134), suggesting that women derive more benefits from peer support in enhancing self-worth. Regarding self-efficacy, both genders exhibit significant positive correlations with social support from parents and educators (*p* < 0.001); however, parental impact is more pronounced for women (0.611) compared to men (0.339), whereas educator support is more robust for men (0.611). Peer support is substantial for women (0.396, *p* < 0.001) but not for men (0.065, *p* = 0.102). In terms of intrinsic motivation, educator support exerts the most substantial influence on both genders (0.719 for men and 0.521 for women, *p* < 0.001), whereas parental support is significant for females (0.529, *p* < 0.001) but not for men (0.109, *p* = 0.093). Peer support influences both males (0.298, *p* = 0.019) and women (0.309, *p* < 0.001), with a more pronounced effect on women. Concerning MPA, all variables, with the exception of peer support, exhibit significant correlations with reduced MPA for both genders (*p* < 0.001). The strongest associations are observed with self-worth (−0.568 for men, −0.621 for women), self-efficacy (−0.383 for men, −0.662 for women), and intrinsic motivation (−0.586 for women, −0.266 for men). Peer support exerts a substantial negative influence on MPA for women (−0.568, *p* < 0.001) but not for men (−0.105, *p* = 0.109), suggesting that peer relationships offer more protective benefits against anxiety for female vocal students. The findings indicate that social support from educators is essential for men, whereas women derive greater advantages from a harmonious blend of support from parents, peers, and educators in regulating self-efficacy, self-worth, and MPA.

The examination of indirect effects indicates notable gender disparities in the impact of social support on MPA via mediators such self-efficacy, self-worth, and intrinsic drive. The most substantial indirect effect on MPA for men is identified through the relationship between educator social support and self-worth, with a significant estimate of −0.371 (*p* < 0.001), indicating that educator support successfully alleviates anxiety by bolstering self-worth. The relationship between educator support and self-efficacy demonstrates a notable indirect effect on MPA (−0.234, *p* = 0.021). Nonetheless, alternative channels, including social support from peers or parents, typically exhibit smaller and frequently non-significant benefits, especially when mediated by self-efficacy or intrinsic motivation. The indirect effect of peer support on self-worth is non-significant (−0.056, *p* = 0.209), suggesting minimal impact on anxiety reduction for men.

Conversely, women demonstrate more significant and persistent indirect effects across several pathways. The relationship between parental support, self-worth, and MPA demonstrates a substantial and robust indirect effect (−0.350, *p* < 0.001), suggesting that parental support significantly alleviates anxiety by enhancing self-worth. Educator support exerts a notable indirect influence on MPA via self-worth (−0.271, *p* < 0.001) and self-efficacy (−0.271, *p* < 0.001), underscoring the critical role of educators in melding these psychological constructs. Furthermore, peer support significantly affects MPA via self-worth (−0.273, *p* < 0.001) and self-efficacy (−0.262, *p* = 0.016), indicating that peers are more essential in alleviating performance anxiety for women compared to men. The indirect effects via intrinsic motivation reveal considerable gender disparities; these pathways are predominantly non-significant for men, whereas women demonstrate significant declines in moderate physical activity (MPA) when parental support (−0.310, *p* < 0.001) and educator support (−0.305, *p* < 0.001) are mediated by intrinsic motivation. The data indicate that females derive advantages from a broader array of social support sources and mediating factors in alleviating MPA, while men predominantly depend on educator support and self-esteem to cope with their nervousness.

### 4.3 Multigroup analysis

Table 9 presents the results of multigroup SEM to assess whether gender moderates the relationships between various forms of social support, psychological variables, and MPA. Significant moderation effects (*p* < 0.05) were found across multiple pathways, suggesting that gender plays a critical role in how social and psychological factors interact. Notably, parental support had a significantly stronger effect on self-efficacy and intrinsic motivation among female students than among male students. In contrast, educator support had a stronger effect on self-efficacy, self-worth, and intrinsic motivation among male students. Peer support had significant gender differences across most paths—females showed stronger associations with self-efficacy and self-worth, while intrinsic motivation remained consistent between genders. Additionally, key psychological predictors, such as self-efficacy and intrinsic motivation, showed significantly stronger negative effects on MPA among females. These findings suggest that female vocalists may be more sensitive to social and motivational factors, underscoring the need for tailored support strategies in music education programs tailored to student gender.

**Table 9:** Multigroup moderation analysis.

Pathway	Male *β*	Female *β*	Δχ^2^ (df = 1)	*p*-value	Moderation significant?
Social support parents → Self-efficacy	0.339	0.611	6.532	0.011	Yes
Social support parents → Self-worth	0.378	0.563	3.822	0.051	No
Social support parents → Intrinsic motivation	0.109	0.529	11.672	0.001	Yes
Social support parents → MPA	−0.498	−0.309	3.202	0.073	No
Social support educators → Self-efficacy	0.611	0.409	5.019	0.025	Yes
Social support educators → Self-worth	0.653	0.437	4.793	0.029	Yes
Social support educators → Intrinsic motivation	0.719	0.521	6.405	0.013	Yes
Social support educators → MPA	−0.618	−0.346	5.107	0.024	Yes
Social support peers → Self-efficacy	0.065	0.396	8.731	0.003	Yes
Social support peers → Self-worth	0.098	0.439	9.654	0.002	Yes
Social support peers → Intrinsic motivation	0.298	0.309	0.127	0.722	No
Self-efficacy → MPA	−0.383	−0.662	7.022	0.008	Yes
Self-worth → MPA	−0.568	−0.621	0.492	0.483	No
Intrinsic motivation → MPA	−0.266	−0.586	5.938	0.015	Yes

## 5 Discussion

The main aim of this study was to examine how different sources of social support, specifically from parents, educators, and peers, influence key psychological constructs, including self-efficacy, self-worth, and intrinsic motivation, and how these constructs affect MPA among vocal music students. Grounded in the SOR framework, SDT, and SCT, the research also explored the mediating roles of psychological variables and the moderating effect of gender in these relationships. The study aimed to comprehensively understand the psychological pathways contributing to MPA in music education contexts by developing and validating a structural model. The results reveal essential direct and indirect effects among the variables, highlighting gender-specific patterns. These findings contribute novel insights into how external support systems and internal psychological resources interact to shape performance-related anxiety in student vocalists.

We particularly compared gender differences to the effect of the interactions among social support, psychological characteristics, and MPA in vocal music students. Such a comprehensive method not only addressed an essential gap in the literature but also provided a good deal of new insight into how different types of social support and psychological constructs may interact to increase or decrease MPA among vocal students, far from trivial, as these results suggested starkly different issues experienced by each gender.

This study makes important additions to MPA research firstly by showing strong negative associations between social support and MPA for males and females vocal music students. The findings show that increased support from parents, educators and friends is significantly associated with lower anxiety about performing in front of others besides music regardless of gender. This aligns with prior studies such as Zarza-Alzugaray et al. (2020), who found that perceived social support plays a crucial role in mitigating performance-related stress in musicians. Similarly, Deci and Ryan (2013) emphasized that supportive interpersonal relationships foster psychological safety, which in turn lowers anxiety and enhances motivation. The current study extends this literature by focusing specifically on vocal music students—a population uniquely vulnerable due to the embodied and exposed nature of voice performance. The consistent negative correlation between all forms of social support and MPA supports the idea that such support can function as a psychological buffer against performance-related stress, in line with Forbes et al. (2025) study. If singing in front of others causes anxiety because it feels personal and exposing, then having support from others—like parents, teachers, or friends—can help reduce that anxiety. This kind of support acts like a shield, protecting people from the stress that comes with performing. The detrimental associations of performance outcome expectations with men’s model and women’s model results suggests the importance of anxiety buffering effects of a supportive social context. It also encourages educators and families to develop such support to help students cope with anxiety better.

Social Support Parents: Addressing MPA involves the multifaceted role parents take to support their young artists; emotional, motivational and practical assistance. The findings of this study align with previous research highlighting the crucial role of emotional support from parents in reducing MPA. Specifically, our results demonstrate that higher levels of perceived parental support are significantly associated with lower anxiety levels among vocal music students. This supports the assertion by Kenny and Holmes (2018) that emotionally supportive parenting fosters a psychologically safe environment where children can express their fears and anxieties without fear of judgment. Such an environment not only destigmatizes emotional disclosure but also normalizes conversations about internal struggles, thereby equipping students with healthier coping strategies. Additionally, our findings echo the mechanisms described by Huang and Yu (2022), who emphasize the value of normalization in reducing anxiety and encouraging emotional resilience in young musicians. By providing consistent emotional support, parents help their children recognize that anxiety is a common experience, thus facilitating the adoption of effective coping mechanisms and promoting psychological readiness for performance. This shift away from fearing failure and towards appreciating the music will hopefully alleviate pressure and thereby reduce performance anxiety. Moreover, parents purposefully assist by creating optimal practice conditions in their home, showing general practice consistency, and organizing logistical aspects of performance like knowing when and where to travel to one (Barnes et al., 2016). This kind of practical help lightens the physical load on young musicians as well as easing anxiety by reducing some external pressures related to performance preparation, making it easier for students to focus on their music.

Social support educators: Educators and music educators are important forecasters of how students approach their performance, manage anxiety, and maintain self-esteem and confidence. The way education is provided, and feedback plays a great role in this regard: positive feedback with an emphasis on development and learning opportunities, not just comments catering to criticism can enhance the sense of self-efficacy and decrease anxiety (Tahirbegi, 2022). Educators not only convey technical skills but also provide students with crucial coping mechanisms to manage performance-related stress. This encompasses methods for addressing the physiological manifestations of anxiety, like breathing exercises, and cognitive strategies such as positive self-talk and mental imagery, which assist pupils in reframing negative ideas and enhancing resilience (MacAfee and Comeau, 2023). Moreover, by establishing low-pressure performance opportunities, educators enable students to rehearse in front of an audience within a supportive environment, thereby enhancing their comfort and confidence on stage. These experiences assist students in adapting to the performance setting, hence diminishing performance anxiety over time (Barros et al., 2022).

Social Support Peers: Peers can profoundly affect a musician’s feeling of performance anxiety, exerting either a beneficial or detrimental influence. The results of this study also align with previous research demonstrating the importance of supportive peer relationships in reducing music performance anxiety. Our findings indicate that stronger peer support is associated with lower levels of anxiety among vocal music students, reinforcing the view that encouragement, shared experiences, and empathetic understanding among peers foster a sense of camaraderie that helps mitigate feelings of isolation and stress (McGrath et al., 2016). This pattern is consistent with the observations of Biasutti and Concina (2014), who noted that when musicians realize their peers face similar performance-related challenges, the experience of anxiety becomes normalized. Such normalization not only reduces the stigma surrounding anxiety but also promotes the sharing of effective coping strategies and mutual emotional support, further contributing to reduced anxiety levels. In ensemble performance contexts, such as orchestras or bands, a supportive peer environment fosters collaboration and reduces judgment, so mitigating the need for perfection and enhancing performers’ comfort and confidence on stage (Schletter, 2020). In contrast, detrimental peer interactions characterized by competitiveness or criticism might intensify anxiety, complicating artists’ ability to handle stress efficiently (Yoder, 2022). Consequently, fostering a pleasant and supportive peer culture in musical environments is essential for alleviating performance anxiety and improving the entire experience for young musicians.

Secondly, the study contributes significant data to the literature about self-efficacy, self-worth, and intrinsic motivation in relation to MPA among vocal music students. Self-worth significantly impacts MPA, since it embodies an individual’s perception of personal value and belief in their inherent worth, irrespective of their accomplishments. Vocal music students with elevated self-worth are less prone to experience severe anxiety prior to performances, as they do not exclusively associate their self-value with their on-stage success. That stability in self-worth protects us from the fear of failure and negative feedback. The most salient negative correlate to MPA, self-worth decreases with anxiety among vocal music students for both men and women. This negative relation means that student who feels confident regarding their inherent worth can deal with performance demand better. On the flip side, students with lower levels of self-worth are more vulnerable to higher anxiety as he or she interprets any mistakes or criticism as evidence of their personal failings. Or, therefore, developing a strong self-worth might be one of the keyways to reduce MPA and enhance performance experiences (Xu, 2025).

Self-efficacy: the belief with about one ability to perform a music performance task at some future date is an essential predictor of performance anxiety experience (Spahn et al., 2023). Musicians with elevated self-efficacy experience reduced anxiety due to their increased confidence in their abilities and lower tendencies to worry about feeling incompetent or that they will receive negative feedback. When that confidence comes from a little past, praise from educators and peers, and good preparation, it is simply a solid belief in self. Musicians with high self-efficacy are more likely to see difficult performances as opportunities to demonstrate their abilities instead of fearsome threats. This attitude minimizes anxiety by creating a sense of reassurance and control over the performance environment (MacAfee and Comeau, 2020). By contrast, low self-efficacy musicians experience increased anxiety due to the likelihood of expecting failure, becoming focused on the potential for error, and feeling pressured to perform at a high standard. Their beliefs in their own abilities are going to be low which perpetuates the anxiety associated with preparing and performing, therefore, making self-efficacy critical within managing performance stress.

Intrinsic motivation: Intrinsic motivation which is the internal drive to engage in music for the pleasure and fulfilment that it brings, may have a significant influence on MPA. Naturally motivated vocal music students tend to approach performance with a focus on the enjoyment and personal satisfaction that singing provides, which reduces the influence of external expectations or fear of negative evaluation. This shift in focus helps lower stress levels, as students place less emphasis on avoiding failure and more on engaging with the performance experience itself. Within men and women, intrinsic motivation greatly lowers MPA; as their levels of intrinsic motivation increases, relatively, you can see a decrease in anxiety. This opposite relationship suggests that those who are driven by a genuine love for music are better able to cope with the performance-related stressors they face. In contrast, students without internal motivation may feel very anxious because they are less involved in the music and more focused on other things like impressing an audience or winning a competition. Therefore, one of the greatest ways to prevent performance anxiety in students would be to foster intrinsic motivation within them, not extrinsic motivation or validation from an educator or competition winners.

Third, this study confirmed, through a theory-driven structural model, that self-efficacy, self-worth, and intrinsic motivation serve as mediators in the relationship between social support of family, peers, educators and MPA in vocal music students. These results align with prior theoretical assertions rather than exploratory discovery. Findings suggest the influence of perceived social support from parents, educators and peers on reduced MPA is indirect, operating through psychological variables. Social support enables students to feel more positive about themselves and motivated from within, and in turn this lowers their anxiety when performing through music. An explorative mediation model provides an account of the interplay between social and psychological factors contributing to performance anxiety in vocal music students, highlighting personal beliefs and goals as key mediators.

There is evidence that self-efficacy mediates the connection between social support and MPA, in that feeling supported by others boosts confidence about the individual talents of music students (Huawei and Jenatabadi, 2024). This is important for vocal music students because vocal performance often requires a high degree of self-expression and therefore vulnerability, where self-efficacy plays an important role in this process. Support from parents, educators, and peers helps nurture a student’s belief that they can succeed by providing them with encouragement, feedback and validation (Shengyao et al., 2024). The result is bolstered self-efficacy, causing students to see difficult things not as impossible but rather something they can tackle, which in turn calms nerves. By way of example, when educators provide constructive feedback, this not only enhances students’ skills but also strengthens their self-efficacy making them less vulnerable to anxiety in high pressure situations (Shengyao et al., 2024). Correspondingly, emotional support from parents and involvement of peers is positively related to a sense of confidence which helps the students to regulate their anxiety better.

The finding that self-worth and intrinsic motivation are additional mediators in the social support; MPA relationship is novel to this study and has not been explored extensively in previous music performance literature. This relationship is moderated by self-worth, the sense of feeling valuable (external control) and influencing how pupils respond to social support. The social support network the vocal music students derive is crucial in nurturing positive self-esteem and subsequently minimizing this susceptibility to anxiety (Perrine et al., 2024). They are secure with themselves as singers and no longer need validation from performance results. Parents who are supportive, and value effort / improvement more than achievement, enable children to experience higher self-worth, which acts as a buffer against anxiety resulting from fear of failure or negative evaluation during performance (Barkela et al., 2024).

Intrinsic motivation would play a mediating role between important social support and MPA because it compels students to engage in music for the inherent pleasure and satisfaction it provides, rather than for external reward or validation. Describing the importance of vocal music students feeling a sense of support when they or their peers have access to some, about those feeling compelled to create something that comes from within them and doing things for its own sake over outside forces influences intrinsic motivation (Hu, 2024). This built-in motivation shifts focus away from anxiety inducing components of performing, like audience members critiquing or competition with other performers, and towards an appreciation for the performance. Accordingly, students who behave with intrinsic motivation experience performance anxiety better because they are purposely motivated to engage in the enjoyable feelings of vocalizing and not for external factors (Oliveira et al., 2024). These results highlight the importance of self-worth and intrinsic motivation as key mediators in the association between social support and MPA, offering new perspectives on the psychological mechanisms that help vocal music students cope with their MPA.

Finally, the findings of this study showed that among Chinese vocal music students, social support has a stronger effect on male students than female ones in terms of self-efficacy, self-worth and intrinsic motivation (shown through multigroup analysis). Even in the case of self-efficacy, self-worth and intrinsic motivation as predictors of MPA, their effects are stronger among females than males.

These interactions were dissimilar for men and women and may be due to the way social support by each gender is integrated and used regarding vocal music (Cui and Chen, 2024). Psychological facets such as self-efficacy, self-worth, and intrinsic motivation may shape to a lesser extent for women when compared to men as social support from parents, educators and peers diagrammatically easily interpolates into the men’s sphere being an influential driver that enhances confidence attributes which the characteristically rely more upon external validation for catalyzing. In many cultures, China included, men are often trained to go after recognition and support from adults or peers, possibly explain why the positive feedback helps heighten these factors (Shi et al., 2024). Positive reinforcement by educators, for example, can quickly raise men’ confidence in their musical abilities as well as increase the desire to practice and perform. And men may also see the encouragement and support from their social network as validation of their skills, which in turn could boost their self-esteem and set off a spiral of motivational processes that lead to men playing music for its intrinsic rewards rather than external rewards.

Strong effects of self-efficacy, self-worth, and intrinsic motivation on MPA in women suggest these psychologic variables are especially important for regulating performance anxiety among female students. This may also be due to women’ tendency to internalize fear and self-doubt more than men, suggesting that improvements in perceptions of self and motivation can have significant impact on their anxiety. In the case of female vocal music students, however, aspects like self-worth and self-efficacy might be more closely related to their emotions in performance situations. When well-designed, they provide a better buffer against the stress and anxiety correlated with performance. Furthermore, because women are often more subject to societal pressures related to appearance and performance standards, a sense of intrinsic motivation can help facilitate a shift from external evaluations to enjoying the intrinsic aspects of music. This transition through reduces the impact of anxiety triggers, making self-efficacy, self-worth and intrinsic motivation key components in reducing MPA in females. Thus, while social support does facilitate development of some traits in men, these particular traits have a stronger influence on reducing performance anxiety in women.

## 6 Conclusion

These findings afford a better understanding of influential relationships related to social support, self-concept, intrinsic determination and MPA in elite singing students. Results now highlight that social support, and in particular the absence of negative emotional influence from parents, teachers/educators and peers, are crucial for psychological outcomes that impact anxiety during performances. The findings of this study support the mediating role of self-efficacy, self-worth, and intrinsic motivation in the relationship between social support and MPA in vocal music, in line with the proposed theoretical model. However, other potential mediators beyond the scope of this study may also contribute to this relationship.

Regarding the gender differences between male and female participants, one of the most important variable social supports; within that context, a big part of the well-being (self-worth/self-efficacy and intrinsic motivation) was perceived as concrete appearance in this study. Results showed social support has stronger effects on psychological traits in men, whereas the opposite pattern is found for their interrelationships contributing to less MPA in women. This indication that directs reinforcement of self-efficacy through social support might benefit men more than women also suggests that enhancing self-worth and intrinsic motivation is more important in reducing anxiety among women. These gender-specific pathways suggest the need for tailored interventions be employed to address MPA, as men and women internalize social support and psychological variables via different routes.

To conclude, the study highlights the need for targeted interventions that incorporate social, psychological, and motivational aspects of management to reduce MPA in singers. Policies that enhance social support systems and respond to sex-specific needs of students are worth consideration by educators, politicians, and university administrators. Through a focus on increasing self-esteem, self-efficacy development and the nurturing of intrinsic motivation, music education programs have the potential to not only help voice students manage anxiety but succeed in their musical pursuits. These findings build on previous MPA literature indicating that successful treatments must consider individual differences.

### 6.1 Implications

Highly relevant theoretical and practical implications for policymakers, educators, administrators, and students are derived by analyzing the gender differences in how social support from parents, educators, and peers affects MPA as well as self-efficacy, self-worth, and intrinsic motivation acts mediators of these relationships. By recognizing that social support might be perceived and received differently by men and women, more specific strategies can be designed to meet the unique needs of each sex thus leading to an increase in successful anxiety-buffering interventions for vocal music students facing performance anxiety. This comprehensive strategy not only improves the theoretical understanding of gender pathways in MPA but also provides a practical foundation for developing an encouraging environment for all MPA vocal music students to thrive within. There are several important theoretical and practical implications to consider:

#### 6.1.1 Theoretical implications

1. This study demonstrates the relevance and applicability of social support theory, thus contributing to a better understanding of it in music education in general, and vocal music students more specifically. It even highlights the order of magnitude for a lot of social support types (e.g., parents, educators, and peers) on psychological variables like self-efficacy, self-worth, and intrinsic motivation that following associate with MPA. This further strengthens the idea that social support is of benefit to well-being but also plays a significant role in relationship experiences specifically associated with performance among students.
2. The study results indicate that theoretical models of MPA should include mediating variables like self-efficacy, self-worth and intrinsic motivation. By investigating factors that mediate the association of social support and MPA, this study provides a more complete framework including both indirect effects and complex relations among social support, and MPA to enhance our understanding of its mechanisms extending beyond the investigation of direct effects.
3. The outcomes emphasize theoretical significance for understanding the gender-specific nature of MPA through social support and psychological characteristics. This research suggests variability between men and women in both the internalization and application of social support that may lead to differential impact on self-efficacy, self-worth and intrinsic motivation. These sex-specific pathways underscore the need for theoretical frameworks that account for gender as a moderator of social support and performance anxiety.
4. Interestingly, this study also focuses on intrinsic motivation consistent with the SDT that is the model states that intrinsics are driven by essential psychological needs satisfaction such as autonomy competence and relatedness. In summary, results support the use of SDT in music performance through revealing that intrinsic motivation supported by social factors can attenuate anxiety. This highlights the value of the theory in explaining motivation and performance outcomes within specific educational and performance environments.
5. SCT stresses the reciprocal interaction between personal factors, environmental influences and actions. It underscores self-efficacy, or the belief in one’s abilities, as a central mechanism for regulating behavior. By illustrating that social support is an important facilitator of self-efficacy that helps students cope with performance anxiety, this study further corroborates SCT. As such, this theoretical lens suggests that confidence-building behaviors such as observational learning, feedback, and reinforcement can reduce anxiety.
6. The combination of these theories (SCT, SDT) can represent an integrated model of these processes which considers interrelations between external stimuli, internal psychological constructs and subsequent action. Integrating aspects of the situate-oriented approach, cognitive and behavioral processes (SCT), and motivational factors (SDT) described above, this study provides a comprehensive account of the mechanisms through which social support may be associated with psychological mediators, such as self-efficacy, self-worth, and intrinsic motivation to decrease MPA. This integration fills in the spaces between theoretical domains and shapes a frame of reference for comprehension of the many factors that contribute to music performance anxiety, opening the doors for more efficacious interventions in the educational and therapeutic realms.

#### 6.1.2 Empirical implications

1. The findings demonstrate that while social support contributes to self-efficacy, self-worth and intrinsic motivation, the impact of these factors is different from males and females indicating the need for specific preventative measures according to gender. There is empirical support suggesting that men gain greater advantages from social support in terms of gains in self-efficacy, while women also appear to benefit more by showing a decrease in anxiety through increases in self-worth and intrinsic motivation. This suggests that tailored approaches are important in the development of programs to target MPA, with interventions specifically targeted to the psychological needs of male and female vocal students.
2. The results highlight the importance of comprehensive support systems within music education that include social, psychological and motivational aspects. According to an empirical data, MPA is affected by numerous psychological factors such as self-worth, perceived competence and intrinsic motivation, which are also known to be influenced by social support from parents, educators and friends. Hence the need for a holistic support program that simultaneously targets these inter-related areas to optimally reduce instrumental anxiety in this population of vocal music students.
3. Results indicate that the source of social support often has a unique effect on adolescent psychosocial characteristics and anxiety levels- parents, educators, peers all exert independent influence. We know from empirical evidence that educator support has stronger effects on men’ self-efficacy and women’ self-worth and intrinsic motivation are more strongly influenced than men’ by peer and parental support. Such findings suggest that music education programs should focus on fostering supportive relationships within multiple social environments while recognizing the unique functions that each source of support has in reducing anxiety.
4. The results demonstrate that self-efficacy, self-worth, and motivation predict from both social support to MPA, but they act as mediators in the correlation between them. This means that music training must focus on these psychological traits as part of the music curriculum. Research provides evidence for embedding workshops or modules in coping, self-efficacy and motivation within music education to equip students with the psychological skills necessary to cope with performance anxiety.

### 6.2 Limitations of the study

1. This limitation of using a sample from a certain culture and educational context in China means that the study’s sample may not represent all vocal music students as they need for more aspects. This limits the generalizability of the results to different cultural contexts (or music education systems) in which social support dynamics and performance anxiety experiences might differ.
2. Cross-sectional studies measure data at one point in time. Because of this approach, it would be impossible to assess changes over time or examine causal relationships between social support and some psychosocial factors with MPA.
3. Social support, self-efficacy, self-worth, intrinsic motivation and MPA could be measured using only questionnaire data that is subject to both social desirability bias and error in individual judgement which may impair the quality of our results.
4. The study focused solely on three forms of social support (parents, educators, and peers) Underestimation of alternative sources of support (mentors, counsellors or extended family members) limited the understanding of the broader network of influences on MPA.
5. Although the measurement tools used in this study were adapted from validated scales, some modifications were made to suit the vocal performance context. These changes, while necessary to improve contextual relevance, may have influenced the internal consistency and construct validity of the measures. Future research is encouraged to re-validate these adapted tools using larger and more diverse samples.

### 6.3 Suggestions for future studies

1. Follow up studies may use longitudinal designs in order to track changes on MPA, social support, self-efficacy, self-worth and intrinsic motivation over time. Such an approach would, over time, allow researchers develop a clearer understanding of causal relationships as well how these might change across a student’s musical development.
2. Broader investigation in vocal music students from different cultural backgrounds and universities would allow for an evaluation of the reproducibility of results across cultures. This could shed light on how culturally different contexts and also the effect of social support structures and other psychological factors may play a role in MPA.
3. Further studies should include alternative sources of social support such as mentors, counsellors, other family members or professional networks, providing broader insights into the multifaceted contributors to MPA.
4. Use both objective measures of MPA and self-reported data in future studies, valid assessment of anxiety can be made through physiological measures (e.g., heart rate, cortisol) or by behavioral observations reactions directly.
5. Follow-up research may evaluate the efficacy of evidence-based interventions targeting self-efficacy, self-worth, and intrinsic motivation in vocal music students. Multiple therapeutic strategies can be tested in research to establish which are the most efficacious ways of ameliorating MPA.
6. Considering the gender differences highlighted in this study, future work should explore gender-specific pathways in more detail and investigate men and women responses to different types of social support. It could lead to the development of programs tailored to each gender’s specific needs.
7. Future research is encouraged to re-validate these adapted tools using larger and more diverse samples. Such efforts would help to confirm the psychometric robustness of the modified instruments in broader populations beyond the current context of vocal music students. Re-validation would also enable researchers to evaluate whether the adapted items maintain their original factor structure, internal consistency, and construct validity across different age groups, musical backgrounds, cultural settings, and performance disciplines.

## Data Availability

The raw data supporting the conclusions of this article will be made available by the authors, without undue reservation.
