# Supplementary material for: Psychological predictors of music performance anxiety among vocal students: a gender-based SEM analysis
Source: Front Psychol. 2026 Feb 2;16:1667730. doi: 10.3389/fpsyg.2025.1667730 (PMC12907393; doi:10.3389/fpsyg.2025.1667730)
Supplement: Supplementary file 1 [file Table_1.DOCX]

1. **Social Support (Ryan et al., 2000) – 5 items each**

**Social Support from Parents**

1. My parents encourage me to pursue my musical goals.
2. I feel emotionally supported by my parents when I perform.
3. My parents show interest in my vocal practice.
4. I can rely on my parents for help with music-related challenges.
5. My parents’ opinions positively influence my confidence in singing.

**Social Support from Educators**

1. My music teacher believes in my singing ability.
2. I feel respected by my vocal instructors.
3. My teachers provide useful feedback on my performance.
4. My educators help me overcome anxiety before performances.
5. I receive motivation from my teachers to perform better.

**Social Support from Peers**

1. My peers support me when I am nervous about singing.
2. I feel accepted by other vocal music students.
3. My classmates encourage me to succeed.
4. I can openly share my singing experiences with friends.
5. I receive positive feedback from my peers after singing.
6. **Intrinsic Motivation (Ryan, 1982) – 24 items across 6 dimensions**

**Interest (4 items)**

1. I enjoy singing for its own sake.
2. I find vocal practice fun and exciting.
3. Singing is one of my favorite activities.
4. I often lose track of time while singing.

**Perceived Competence (4 items)**

1. I think I am good at singing.
2. I feel confident in my singing skills.
3. I believe I can sing complex pieces well.
4. I perform vocal tasks with ease.

**Importance (4 items)**

1. Singing is important to who I am.
2. My identity is closely tied to being a vocalist.
3. Singing is a central part of my life.
4. I take singing seriously.

**Tension (4 items)** *(reverse-coded)*

1. I feel anxious when practicing vocal music.
2. Singing makes me tense.
3. I get nervous even thinking about performing.
4. Vocal music causes me stress.

**Perceived Choice (4 items)**

1. I choose to sing because I want to.
2. I feel free to decide how I practice singing.
3. I enjoy having control over my singing routine.
4. I sing because it’s my personal choice.

**Value (4 items)**

1. Singing has great value in my life.
2. I believe that singing is a worthwhile activity.
3. Singing helps me grow as a person.
4. Singing is beneficial for my future.
5. **Self-Efficacy (Zelenak, 2011) – 24 items across 4 dimensions**

**Enactive Mastery Experience (6 items)**

1. I have successfully sung difficult pieces in the past.
2. My past performances give me confidence.
3. I remember times I overcame challenges in singing.
4. I feel proud of my vocal achievements.
5. I’ve received awards or praise for singing.
6. I can handle new singing tasks well.

**Vicarious Experience (6 items)**

1. Watching skilled singers motivates me.
2. I learn by observing others perform.
3. Seeing classmates succeed boosts my confidence.
4. I model my practice after strong performers.
5. Observing others helps me improve.
6. I get ideas from other vocalists' techniques.

**Verbal/Social Persuasion (6 items)**

1. People tell me I’m a good singer.
2. Encouraging comments make me feel capable.
3. I trust the positive feedback I receive.
4. Compliments improve my singing confidence.
5. I feel more confident after talking to my coach.
6. I believe in myself when others encourage me.

**Physiological and Affective State (6 items)**

1. My body reacts negatively before performances.
2. I get sweaty or shaky before I sing. *(reverse-coded)*
3. I feel calm and in control while performing.
4. I interpret nervousness as excitement.
5. My mood affects how well I sing.
6. Physical signs help me understand my readiness.
7. **Self-Worth (Xu, 2023) – 11 items**
8. I believe I am a valuable person.
9. I feel proud of myself regardless of performance.
10. I think I am a worthwhile individual.
11. I respect myself even when I make mistakes.
12. My self-worth does not depend on others' approval.
13. I have confidence in my personal abilities.
14. I handle criticism without losing self-respect.
15. I am comfortable being myself.
16. I feel deserving of success.
17. I bounce back quickly from failure.
18. I accept myself as I am.
19. **Music Performance Anxiety (MPA) (Kenny, 2009; Papageorgi et al., 2007; Ryan & Andrews, 2009) – 14 items**
20. I feel nervous before singing in public.
21. My hands tremble when I sing in front of others.
22. I worry that I will make mistakes on stage.
23. I avoid opportunities to perform.
24. I forget lyrics under pressure.
25. I feel physical symptoms of anxiety when I sing.
26. I fear being judged while singing.
27. My heart races before I go on stage.
28. I get anxious even during rehearsals.
29. I worry about not meeting expectations.
30. I get butterflies before performing.
31. I doubt my ability before going on stage.
32. My anxiety affects my vocal control.
33. I struggle to relax before a performance.
